# Terminal hairpins improve protein expression in IRES-initiated mRNA in the absence of a cap and polyadenylated tail

**DOI:** 10.1038/s41434-023-00391-4

**Published:** 2023-02-24

**Authors:** Victor Solodushko, Brian Fouty

**Affiliations:** 1 Department of Pharmacology, University of South Alabama School of Medicine, Mobile, AL 36688 USA; 2 The Center for Lung Biology, University of South Alabama School of Medicine, Mobile, AL 36688 USA; 3 Department of Internal Medicine, University of South Alabama School of Medicine, Mobile, AL 36688 USA; 4 The Division of Pulmonary and Critical Care Medicine, University of South Alabama School of Medicine, Mobile, AL 36688 USA

**Keywords:** Translation, RNA vaccines

## Abstract

Synthesizing mRNA in vitro is a standard and simple procedure. Adding the 5′ cap and 3′ polyadenylated (poly(A)) tail to make this mRNA functional for use as a vaccine or therapy increases the time and cost of production and usually decreases the yield, however. We designed mRNA that lacked a cap and poly(A) tail but included an internal ribosomal entry site (IRES) to initiate protein translation. To protect the 5′ and 3′ ends of mRNA from exonucleases, we added stable terminal hairpins. When compared against typical mRNA (i.e., mRNA that contained a cap and poly(A) tail but lacked hairpins), expression of the delivered reporter protein in HEK293 cells was similar. Using a triple instead of a single hairpin at each end increased protein expression even more. This method has the potential to simplify the production and reduce the cost of synthesizing exogenous mRNA for use as biologics or vaccines.

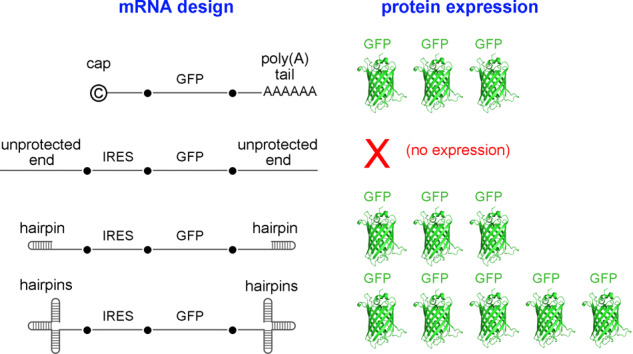

## Introduction

Both *endogenous* mRNA which is produced in the nucleus and then transported to the cytoplasm, and therapeutic *exogenous* mRNA which is delivered to cells as a biologic agent are rapidly degraded by the same mechanisms. This makes exogenous mRNA attractive for use as a therapeutic agent where only short-term expression of a protein is required, such as for a transient disease/injury or as a vaccine. mRNA-based vaccines were the first vaccines against SARS-CoV-2 approved in the United States and induce both humoral and cell mediated immunity against its spike protein [1]. A major advantage of such vaccines is that mRNA can be produced in the laboratory or a GMP facility from a DNA template using readily available materials, less expensively and faster than conventional vaccines such as subunit, live-attenuated, and inactivated viruses; the use of mRNA vaccines also avoids safety issues intrinsic to working with live viruses. This permits simpler downstream purification and rapid manufacturing [2].

Almost all endogenous eukaryotic mRNA contains a 5′ cap structure and a 3′ chain of adenosine nucleotides (poly(A) tail) added during RNA processing. The 5′ cap (an N7-methylated guanosine linked to the first nucleotide of the RNA via a reverse 5′ to 5′ triphosphate linkage (the 5′ m^7^G cap)) [3, 4] is critical in initiating protein synthesis, but also functions as a protective group against 5′ to 3′ exonucleases; [3, 5–10] the poly(A) tail increases translational efficiency and improves message stability by protecting against 3′ to 5′ exonucleases [11–15]. mRNA can be synthesized in vitro using prokaryotic phage polymerases, such as T7, T3, and SP6 [16–19]. The 5′ cap can be incorporated into mRNA during transcription [20–24] or it can be added after transcription [3, 4, 25–28]; the poly(A) tail can be directly encoded within the DNA template [11, 29, 30] or it can be added after transcription [31–33].

Both the capping and the addition of the long poly(A) tail are critical for proper function and stability of the transcribed mRNA, but both slow production and add cost. Redesigning mRNA so that they retain their function and stability without the requirement for a cap and a lengthy poly(A) tail would reduce the cost, increase the speed, and improve the yield, of in vitro mRNA production.

In 1988, Jang discovered that encephalomyocarditis viral (EMCV) RNA is translated by a distinctly different mechanism in which ribosomes initiate translation on highly structured regions of RNA located within the 5′-UTR [34]. These regions were named internal ribosome entry sites (IRESs). Since then, numerous IRESs have been identified in other viruses as well as in eukaryotes. It is speculated that more than 10% of all human genes may be translated via IRESs, although only a small fraction of these have been identified and characterized [35].

IRES sequences are widely used in molecular biology to co-express several genes under the control of the same promoter. They are often included in bicistronic constructs where the IRES segment is located between two open reading frames (ORF) in an mRNA molecule; this allows translation of the downstream protein coding region independently of the 5’ cap of the mRNA molecule [36]. In such a setup, both proteins are produced in the cell; the first protein, located in the first cistron, is synthesized by cap-dependent initiation, whereas translation initiation of the second protein (in the second cistron) is directed by the IRES element located in the intercistronic spacer [37]. And while a poly(A) tail seems to be important for mRNA stabilization in cases where the EMCV IRES is used, it appears to be less vital for translation initiation [38].

The idea of synthesizing an mRNA vaccine that harbors only an IRES on the 5′ end without the need to add a cap and a long poly(A) tail is attractive due to its simplicity. The main drawback of such an approach, however, is the extreme sensitivity of both the 5′ and 3′ ends of unprotected mRNA to exonucleases making it unstable and highly degradable.

While almost all endogenous eukaryotic mRNA contains a modified 5′ cap structure, in prokaryotes, the 5′ end of the newly transcribed mRNA is not further modified and retains the 5′ triphosphate. Instead of a cap, it has been shown that *Escherichia coli* protects its 5′ end from prokaryotic exonucleases by forming a 5′-terminal stem-loop structure [39]. The presence of this 5′-terminal stem-loop, formed by the pairing of complementary nucleotide base pairs, can prolong the life of mRNA by as much as a factor of 5 presumably because prokaryotic exonucleases have problems initiating degradation close to stable stem structures [40, 41].

We used this strategy to protect the 5′ and 3′ ends of exogenously produced mRNA. First, we generated mRNA harboring the EMCV IRES within its 5′ UTR and demonstrated that it could initiate translation of a downstream ORF (the reporter eGFP) without a 5′ cap and poly(A) tail. However, the expression was very low. Therefore, this mRNA was then modified to form stable hairpin structures on both ends of the mRNA to bury vulnerable unmodified ends within the structured mRNA molecule to protect it against exonucleases. We compared the ability of this mRNA to express eGFP in eukaryotic cells against more conventional mRNA constructs (i.e., containing a 5′ cap and a 3′ poly(A) tail without hairpins). We showed that when stable hairpin structures are present on both ends of mRNA, the eGFP expression increased more than 36 times compared to mRNA without these hairpins and was similar to classical mRNA that contained a cap and a long poly(A) tail. Using triple rather than single hairpins at each end improved eGFP expression in target cells even more.

The mRNA transcripts described here can be produced in vitro in a single step for use as therapy or as a vaccine without the need for capping and polyadenylation.

## Materials and methods

### Materials

Dulbecco’s Modified Eagle Medium (DMEM), Opti-MEM, 0.05% trypsin/0.53 mM EDTA and L-glutamine were all purchased from Gibco (Grand Island, NY). Fetal bovine serum (FBS) was purchased from Atlanta Biologicals (Lawrenceville, GA). Gentamicin Sulfate was from Corning (Corning, NY). All restriction enzymes, DNA polymerase I (Klenow), T4 DNA ligase, Deoxynucleotides, Poly(A) polymerase, 3′-O-Me m^7^G(5′)ppp(5′)G RNA cap structure analog, High Efficiency Competent *E. Coli* Cells [NEB 10-beta] and HiScribe™ T7 Quick High Yield RNA Synthesis Kit were from New England BioLabs (Ipswich, MA). Hi-Lo DNA Markers were obtained from Minnesota Molecular, Inc. (Minneapolis, MN). QIAprep spin miniprep kit was from Qiagen (Germantown, MD). LB Broth was purchased from Alfa Aesar (Ward Hill, MA). N^1^-Methylpseudouridine was from TriLink Biotechnologies (San Diego, CA). TransIT-mRNA lipid transfection kit was from Mirus Bio (Madison, WI). CellTiter 96® Aqueous One Solution Cell Proliferation Assay was from Promega (Madison, WI). Ethidium homodimer was purchased from Molecular Probes (Eugene, OR).

### Vectors

All template plasmids were derived from the pBR322 plasmid backbone (New England BioLabs, NEB# N3033) and contained a prokaryotic origin of replication and an ampicillin resistance gene for selection and were amplified in *E. coli* cells (NEB 10-beta, New England BioLabs, NEB# C3019H) in LB Broth (Alfa Aesar, H26676) at 37 ^o^C overnight. The plasmids were then isolated from *E. coli* and purified using QIAprep Spin Miniprep Kit (Qiagen, Cat. No. 27106) following the manufacturer’s protocols. DNA plasmids were linearized by the BsaI-HF®v2 restriction enzyme (New England BioLabs, NEB# R3733S) to terminate T7 polymerase and used as templates for a T7 promoter driven in vitro RNA synthesis.

HiScribe™ T7 Quick High Yield RNA Synthesis Kit (New England BioLabs, NEB# E2050 and NEB#E2040) was used to generate up to 180 μg of RNA per reaction from 1 μg of each linearized DNA template following DNase treatment to remove DNA template and LiCl Precipitation. For some experiments, mRNAs were synthetized using N^1^-Methylpseudouridine (m^1^Ψ) (TriLink Biotechnologies, N-1081-10) as a substitute for all uridines (U). The synthetized RNAs were used as experimental mRNA vectors. Some mRNAs were designed to form a single stable 5′ (24 paired bp) and/or 3′ (30 paired bp) terminal hairpin (see schematics in figures). All hairpins had about 70% G/C content. Some mRNAs were designed to form a triple stable hairpin with 48, 34, and 36 paired bp individual hairpins on the 5′ end and/or 20, 21, and 28 paired bp individual hairpins on the 3′ end connected immediately to each other with no unpaired nucleotides in between (see schematics in corresponding figures). Each individual hairpin of the triple hairpin is structurally different from the other individual hairpins to avoid potential interference due to the formation of secondary structures.

Some mRNAs included 5’ (40 bp) and/or 3′ (40 bp) short poly(A) sequences that were placed either terminally (in the absence of hairpins) or directly connected to the terminal hairpins. All mRNA vectors encoded eGFP as the reporter protein. Most of the mRNA vectors had an EMCV IRES sequence upstream of the eGFP ORF. In the figures, the names of control vectors that did not have an IRES are underlined. All mRNA vectors with a single hairpin or with unstructured ends (unpaired nucleotides) had the same 5′ (68 bp) and 3′ (104 bp) length UTR, which flanked the internal cassette (IRES-eGFP or eGFP alone in some control vectors); vectors with internal poly(A) stretches had 40 bp longer 5′ and/or 3′ UTR sequences; vectors with a triple hairpin structures had 137 bp 5′ UTR and/or 186 bp 3′ UTR. For an efficient in vitro transcription from the T7 promoter, all vectors with a short terminal 5′ poly(A) segment had two G nucleotides in front of the poly(A) stretch (GGAAAAAA…). The basic internal design of the tested mRNAs is shown in the Graphical Abstract and includes the eGFP coding region and an upstream IRES to initiate translation. To highlight the important differences between the mRNAs while keeping figures as simple as possible, only the differences in the terminal ends are shown. Although the length of the actual mRNA vectors varies, all figures are drawn to similar scale.

Some mRNA vectors were tailed with poly(A) (range from 75–200 adenosines) using *E. coli* Poly(A) Polymerase (New England BioLabs, NEB# M0276). The purity of mRNA and the length of poly(A) tails (if applicable) were confirmed by gel electrophoresis. Some mRNA vectors were capped with 3´-O-Me-m^7^G(5′)ppp(5′)G RNA Cap Structure Analog (also known as Anti-Reverse Cap Analog (ARCA), New England BioLabs, NEB# S1411) using a 6:1 ratio of cap analog to GTP.

### Cells

HEK293 cells (Human Embryonic Kidney cell line [Cat. No. CRC-1573]) and MDCK (NBL-2) cells (Madin-Darby Canine Kidney cell line [Cat. No. CCL-34]) were obtained from ATCC. Cells were grown in humidified incubators in DMEM/10%FBS supplemented with Gentamicin Sulfate and L-glutamine at 37 °C in 5% CO_2_ and routinely passaged after reaching 80% confluency. Cells were harvested by 0.05% trypsin/0.53 mM EDTA digestion and counted with Coulter Z1 (Coulter Electronics).

mRNAs designed for this project are compatible with any method of cellular transfection. However, to exclude any effect of lipid or cationic polymer formulations on mRNA stability and functionality, we chose to deliver mRNA by electroporation. In some experiments, we confirmed our electroporation findings with TransIT-mRNA lipid transfection kit (Mirus, Prod. No. 22024790). For mRNA vector electroporation, HEK293 or MDCK cells were suspended in 200 μl of ice-cold Opti-MEM. Cell suspensions containing 5 × 10^5^ cells were aliquoted into pre-chilled 4 mm electroporation cuvettes (BTX). Ten μg of vector RNA was added into the cuvette. Negative control cells were electroporated in the same way, but no RNA was added. The cuvette was then inserted into the BTX 830 (Holliston, MA) electroporation system and electroporation was carried out using a single pulse at 150 V for 15 milliseconds. The cells were then transferred into a 35 mm cell culture dish containing complete medium and incubated at 37 °C for 24–96 h. Cells were trypsinized and analyzed by BD Biosciences Canto II cell analyzer in the University of South Alabama Flow Cytometry Core.

Cell viability was assessed by CellTiter 96® Aqueous One Solution Cell Proliferation Assay (Promega, G3580), and ethidium homodimer uptake (Molecular Probes, L-3224); cell growth was determined by cell counts over 3 days.

### Statistical analysis

Data are expressed as mean ± SD. Changes in eGFP expression were compared using one-way ANOVA combined with Fisher post hoc analysis, with a *P* < 0.05 considered significant. In all figures ‘n’ represents the number of independent experiments. Each independent experiment was done on a separate day and was done in triplicate.

## Results

### Adding terminal hairpins to IRES-based mRNA vectors increased eGFP expression

**Fig. 1:**
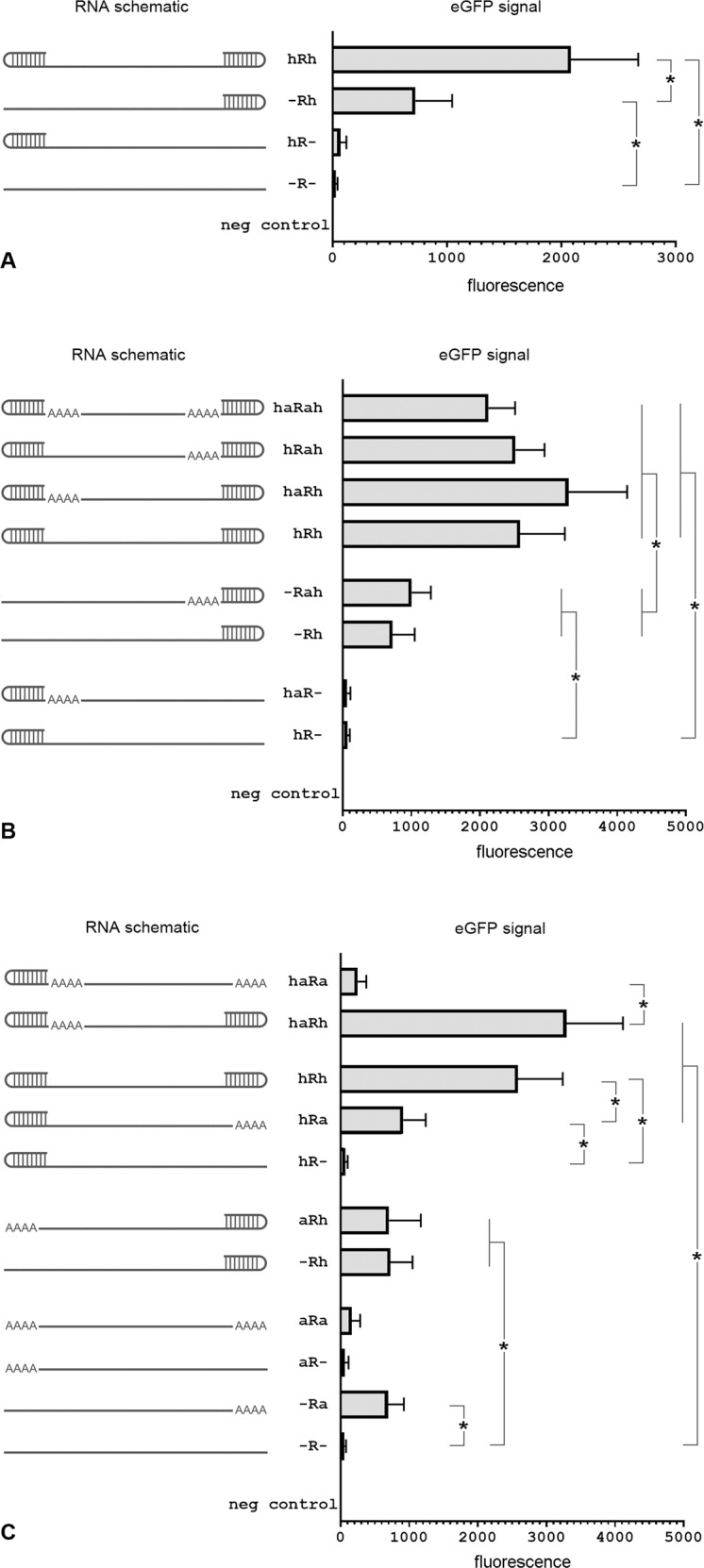
Including hairpins at the terminal ends of IRES-harboring mRNA vectors improves eGFP expression. RNA vector schematics with corresponding eGFP fluorescent signal in transfected HEK293 cells 24 h after electroporation. **A** All four vectors had identical sequences except in their terminal ends that either formed or did not form stable hairpins. **B** All vectors had identical sequences except in their terminal ends that either formed or did not form stable hairpins and included or did not include short internal poly(A) sequences that flanked the 5′ and the 3′ terminal hairpins. **C** All vectors had identical sequences except where terminal hairpins or short poly(A) sequences are indicated. [h: hairpin; a: short poly(A) sequences; R: main internal segment that includes an IRES and the eGFP ORF] (*n* = 4, *P < 0.05).

We first studied the effect of incorporating stable secondary RNA hairpin structure(s) on either or both ends of mRNA on the efficiency of eGFP expression 24 h after vector transfection into HEK293 cells by electroporation (Fig. 1A). The results were compared to eGFP expression from a control vector (-R-) that contained an IRES with the same length 5′ and 3′ UTR but lacked stable terminal hairpin structures. In these initial studies, we constructed mRNA vectors of identical length and internal sequences to control for these factors when interpreting results.

Transfection with the control vector without any hairpins (-R-) resulted in detectable, but very low eGFP expression (Fig. 1A). eGFP expression increased slightly if the vector contained a 5′ hairpin (hR-), but the presence of a hairpin at the 3′ end (-Rh) had a significantly greater positive effect on eGFP expression. Delivery of a vector containing both 5′ and 3′ hairpins (hRh) resulted in the highest eGFP expression. These findings indicate that the addition of terminal secondary structures improved IRES-initiated translation in vectors lacking a cap and a poly(A) sequence.

### Adding short *internal* poly(A) sequences to IRES-based mRNA vectors did not increase eGFP expression

In some pox viruses, the presence of 5′-proximal short poly(A) sequences can act as strong translational enhancers that mediate the formation of the ribosomal initiation complexes independently of some multifunctional eIFs, (eIF3 and eIF4F) [42] potentially adding a new function to a poly(A) stretch if placed in other than a 3′ terminal position. Internal polyadenosine sequences can serve as an additional point of entry for the poly(A) binding protein (PABP) and play an important role in regulating gene expression, especially in IRES-driven vectors that showed much less dependence on the 3′ poly(A) terminal tract for translation initiation [38, 43].

To determine whether internal poly(A) sequences would improve translational efficiency in vectors with terminal hairpins, we inserted relatively short (40 bp) poly(A) stretches immediately downstream of the 5′ terminal hairpin or upstream of the 3′ terminal hairpin and assessed eGFP expression. All vectors had an identical IRES-eGFP internal transcription cassette and differed only by the presence or absence of the terminal hairpins and short internal poly(A) segments. HEK293 cells were electroporated with these mRNA vectors and eGFP signal was measured 24 h later.

eGFP expression was higher in vectors that had both 5′ and 3′ hairpins (Fig. 1B), when compared to vectors that had a hairpin on only one end; differences in eGFP expression among the four vectors with hairpins on each end were non-significant. The presence of poly(A) sequences downstream of the 5′ hairpin or upstream of the 3′ hairpin had little to no effect on eGFP expression compared to similar vectors without internal poly(A) sequences. Consistent with the results shown in Fig. 1A, vectors with a hairpin only at the 3′ end expressed more eGFP than vectors with a hairpin only at the 5′ end.

### Adding short *terminal* poly(A) sequences to IRES-based mRNA vectors did not increase eGFP expression

The addition of a longer poly(A) tail at the 3′ end of mRNA increases the protein expression of capped messengers by stabilizing RNA and activating translation regulatory mechanisms [44]. Whether this is also true with short poly(A) stretches when the vectors initiate translation from an internal IRES is not well studied.

We replaced the 5′ and 3′ terminal hairpins with short poly(A) stretches and determined translation efficiency of these vectors in HEK293 cells. Cells were electroporated with the appropriate vector and eGFP expression was measured 24 h after vector delivery. The addition of a short terminal 3′ poly(A) sequence modestly increased eGFP expression in vectors that lacked a 3′ terminal hairpin (-R- vs -Ra, aR vs aRa, and hR- vs hRa; Fig. 1C), whereas adding a short terminal 5′ poly(A) sequence in vectors that lacked a 5′ terminal hairpin had no significant effect on eGFP expression (aR- vs -R-, aRa vs -Ra, aRh vs -Rh; Fig. 1C). The positive effect of hairpins on eGFP expression was much greater than the effect of short terminal poly(A) sequences. Again, vectors with hairpins at both ends of the vector had the highest eGFP expression compared to vectors with other terminal end configurations.

### Capless and tailless IRES based mRNA vectors with hairpins at both ends had equivalent eGFP expression as canonical mRNA vectors

**Fig. 2:**
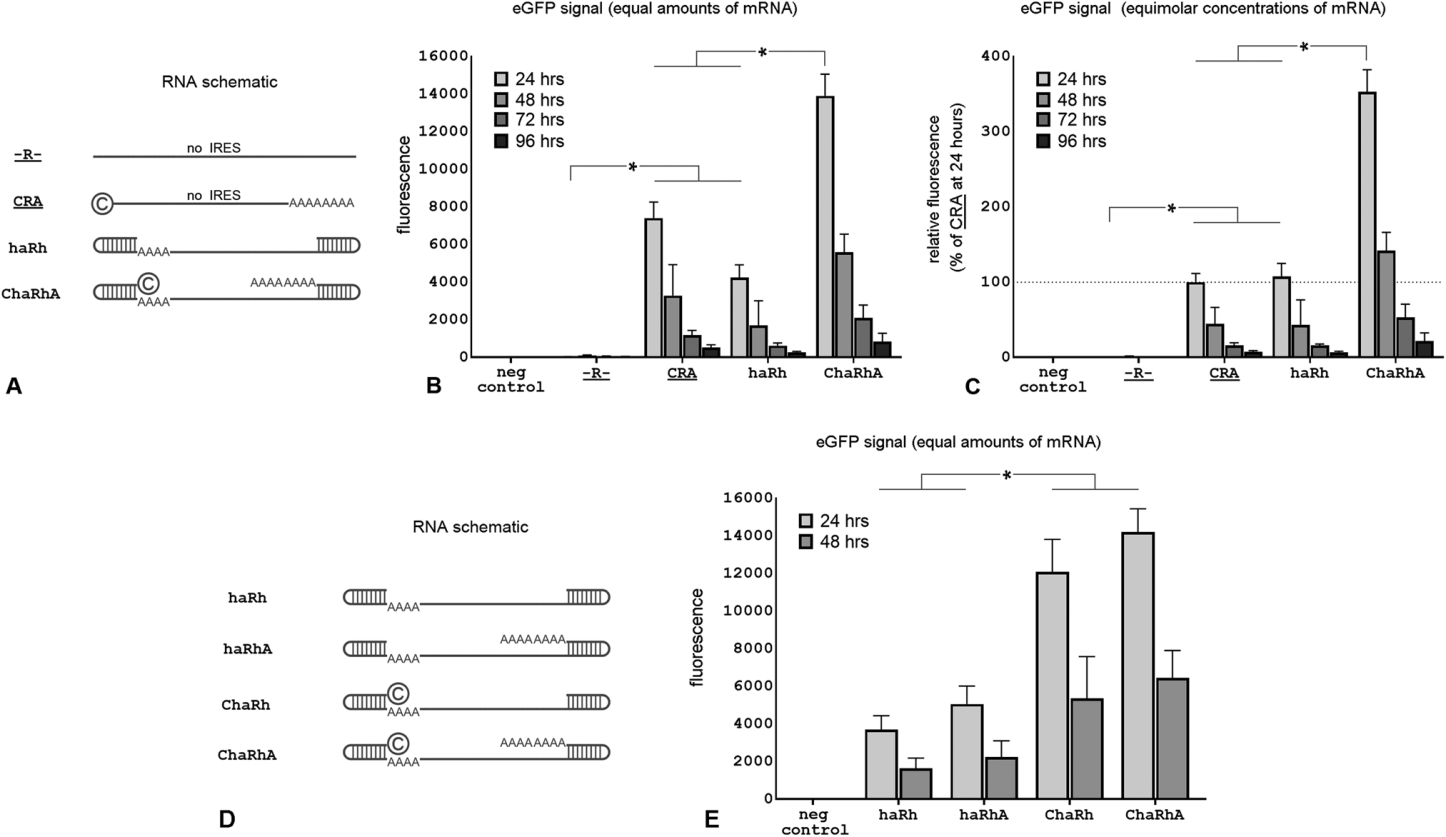
IRES-based mRNA vectors with terminal hairpins demonstrate an equivalent eGFP expression as canonical mRNA vectors. RNA vector schematics with corresponding eGFP fluorescent signal in transfected HEK293 cells at different time points after electroporation. **A** mRNA vector schematics for **B**, **C**. Vectors -R- and CRA had an eGFP ORF but no IRES. Vectors haRh and ChaRhA had a short poly(A) sequence that flanked the 5′ terminal hairpin (AAAA), an IRES, and the eGFP ORF. Vectors CRA and ChaRhA had a cap (©, ARCA) and a long poly(A) tail (AAAAAA). **B** eGFP expression when equal amounts of mRNA were used; and **C** eGFP expression when equal molar concentrations of mRNA were used. **D** mRNA vector schematics for **E**. All vectors were identical except where the 5′ cap (C), or long 3′ poly(A) tail (AAAAAA) are indicated. **E** eGFP fluorescent signal in transfected HEK293 cells 24 and 48 h after electroporation when equal amounts of mRNA were used. Vectors with no IRES are underlined. [h: hairpin; a: short internal poly(A) sequences (AAAA); A: long poly(A) tail (AAAAAA); C: Anti-reverse Cap Analog (ARCA); R: main internal segment that includes the eGFP ORF in non-IRES vectors or an EMCV IRES and the eGFP ORF] (*n* = 4, *P < 0.05 shown for 24 h only).

Capless and tailless IRES based mRNA vectors with single hairpins at both ends had high levels of eGFP expression in our experiments. To determine how their expression compared to conventional (capped and tailed) exogenous mRNA, we generated a vector (CRA) that contained the coding region for eGFP, a 5′ cap, and a 3′ poly(A) tail, but lacked an IRES and was designed to not form terminal hairpins. We compared this vector to vector haRh which showed the best performance in the previous experiment. Cells transfected with an unmodified vector (-R-) (which contained the eGFP coding region, but lacked a 5’ cap, an IRES, and a long terminal poly(A) tail) and cells electroporated without RNA were used as negative controls. We measured eGFP levels in transfected cells between 24 to 96 h after vector delivery (Fig. 2A–C). Vectors CRA (which initiated translation using a 5’ cap and was poly-adenylated) and haRh (which initiated translation through an IRES and lacked a cap and poly(A) tail) had comparable eGFP expression when used in equimolar concentrations (Fig. 2C). Adding a 5’ cap and a 3’ poly(A) tail to the mRNA vector with 5′ and 3’ hairpins (haRh) to form vector ChaRhA further increased eGFP expression beyond that of either vector alone (Fig. 2B, C). To determine which modification was primarily responsible for the increased eGFP expression from the ChaRhA vector (the cap or the long poly(A) tail), we generated two more vectors: ChaRh which was capped but lacked a long poly(A) tail and haRhA which was not capped but had a long poly(A) tail (Fig. 2D). The addition of a cap (ChaRh) significantly increased eGFP expression, whereas the addition of the poly(A) tail (haRhA) had little effect on eGFP expression (Fig. 2E). The effect of adding both a cap and a poly(A) tail was not significantly different than that of adding the cap alone and differences in eGFP expression were significant only between capped and uncapped groups. Similar to the findings in Fig. 2A–C, there was no difference between vectors in the rate of eGFP signal decay over time.

### Adding triple terminal hairpins to IRES-based mRNA vectors further increased eGFP expression

As shown above, RNA vectors with single terminal hairpins are able to support higher levels of eGFP expression in cells than vectors that lack hairpins. This may be due to the resistance of RNA with more secondary structures to degradation by exonucleases [45]. It is possible that the presence of an even more complex secondary structure in close proximity to either end of the RNA may further protect against exonucleases by physically limiting access of the exonucleases to the terminus.

**Fig. 3:**
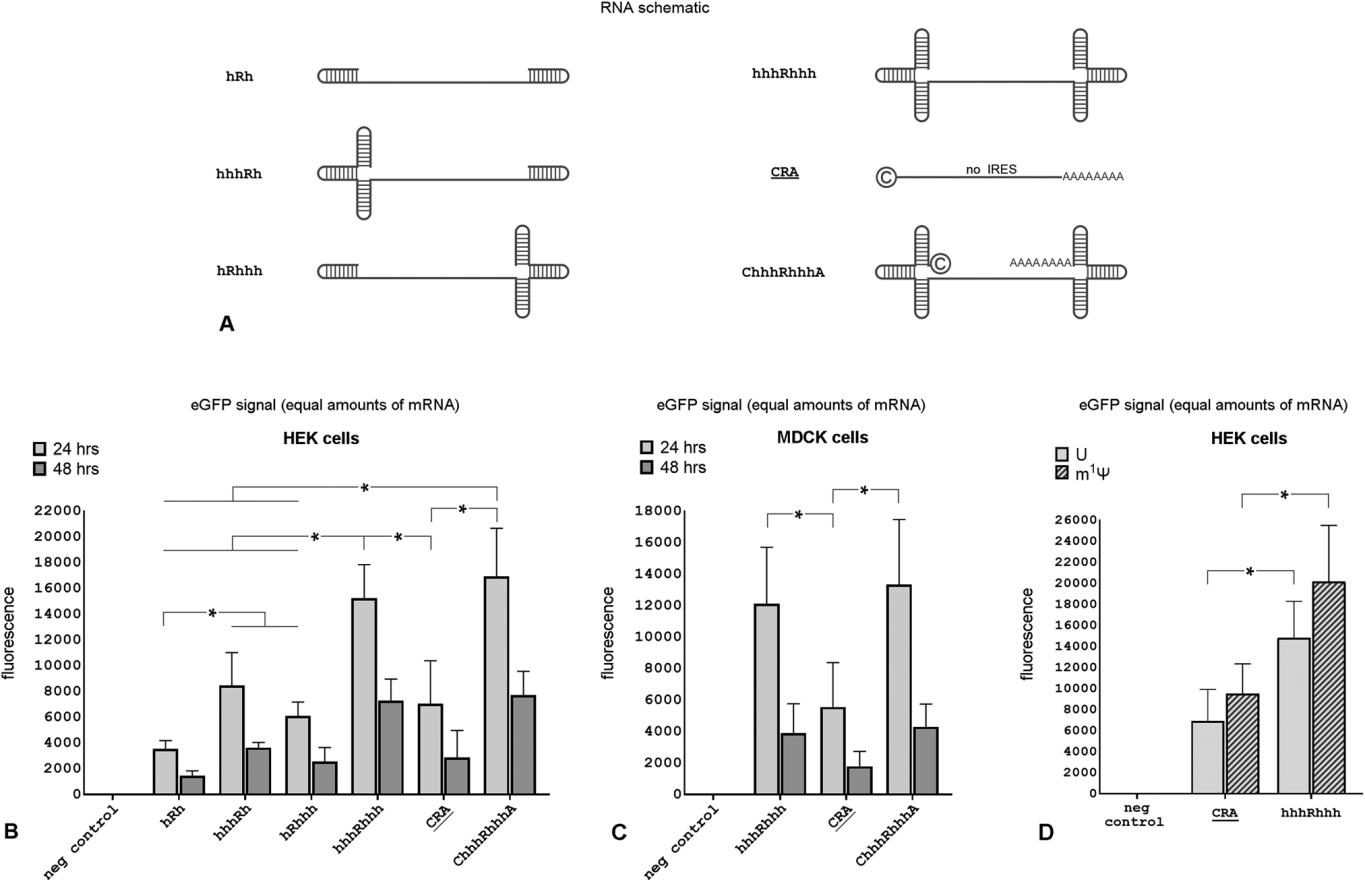
IRES-based mRNA vectors with triple terminal hairpins at both ends had a higher eGFP expression than canonical mRNA vectors. **A** Vector schematics. Vectors with triple hairpin structures had slightly longer UTRs. **B** eGFP expression in HEK293 cells 24 and 48 h after electroporation with equal amounts of the indicated mRNA vectors. Differences between hhhRhhh and its capped and polyadenylated variant ChhhRhhhA were not significant, but both outperformed canonical mRNA (CRA). **C** eGFP expression in MDCK cells 24 and 48 h after electroporation with equal amounts of the indicated mRNA vectors. **D** eGFP expression in HEK293 cells 24 h after electroporation with modified mRNA vectors in which all uridines were replaced by N1-Methylpseudouridine (m1Ψ). [h: hairpin; A: long poly(A) tail (AAAAAA); C: Anti-reverse Cap Analog (ARCA); R: main internal segment that includes the eGFP ORF in non-IRES vectors or an IRES and the eGFP ORF] (*n* = 4, *P < 0.05 shown for 24 h only).

To determine whether including a triple terminal hairpin structure in the delivered RNA improved protein expression above that of a single hairpin, we constructed three new mRNA vectors (without any poly(A) sequences because they had no effect on eGFP expression as shown in Fig. 1B) and compared them to a vector harboring single hairpins on each end (hRh) (Fig. 3A). In one vector we replaced a single 5′ hairpin with a triple hairpin structure (hhhRh). The second vector was constructed by replacing a single 3′ hairpin in the hRh vector with a 3′ triple hairpin structure using the same strategy (hRhhh). The third vector had triple hairpin structures on both ends (hhhRhhh). We compared all these vectors against a canonical mRNA (CRA) and a capped and adenylated version of mRNA with triple hairpins on both ends (ChhhRhhhA).

All vectors with triple hairpin structures demonstrated enhanced eGFP expression compared to the single hairpin vector (hRh) (Fig. 3B). The vector that contained triple hairpin structures at both the 5′ and 3′ ends (hhhRhhh) resulted in the highest eGFP level at both time points outperforming the classical mRNA construct (CRA). Unlike the effect of adding a cap and poly(A) tail to single hairpin constructs (Fig. 2E), however, adding them to the triple hairpin constructs did not lead to a statistically significant increase in eGFP expression (Fig. 3A–C). The fluorescent signal at 48-h post-electroporation was about 42–43% of the value seen at 24-h for all vectors except for the one with triple hairpins at both ends (hhhRhhh) where fluorescence was 48%. The decrease in fluorescent decay rate is likely explained by improved vector stability in cells.

To confirm that using heavily structured ends in IRES-harboring vectors worked in cells other than HEK293 cells, we repeated the experiments in MDCK cells and found similar results (Fig. 3C). To confirm that this strategy also worked if modified ribonucleotides were used, N^1^-Methylpseudouridine (widely used in approved mRNA vaccines [46, 47]) was substituted for uridine without any decrease in vector performance (Fig. 3D). Introduction of these mRNAs into HEK293 and MDCK cells had no detrimental effects on cell viability as assessed by cell proliferation assay, cell growth over 3 days, or cell membrane permeability (data not shown).

## Discussion

Here, we show that mRNA designed to form hairpin secondary structures at both the 5′ and 3′ ends maintains a high level of reporter expression in eukaryotic cells, even in the absence of a 5′ cap and 3′ polyadenylated tail, as long as an internal ribosome entry site (IRES) is included in its 5′ UTR. Equimolar levels of IRES-containing mRNA vectors with single hairpins at both terminal ends showed the same level of protein expression as conventionally constructed (non-IRES-containing) mRNA that contained a 5′ cap and a 3′ poly(A) tail. Using a triple hairpin structure instead of a single hairpin at both ends further increased protein expression, outperforming capped and poly-adenylated vectors without an IRES.

In general, exogenous mRNA is produced in three steps: (1) in vitro mRNA synthesis from a DNA template, (2) the addition of a modified guanosine cap on the 5′ end of the mRNA, and (3) the addition of a poly(A) tail on the 3′ end of mRNA [2, 3, 17]. mRNA may be capped during transcription by including the cap analog in the nucleotide mix during synthesis or the cap can be added after the mRNA is completely transcribed [3, 4, 20–28]. Regardless of the method used, it always results in a fraction of mRNA that is uncapped which renders it translationally inactive [3]. In terms of the poly(A) tail, relatively short poly(A) tails can be directly added to the end of the mRNA during transcription by including the sequence into the DNA template [11, 29, 30]. Alternatively, longer poly(A) tails that result in more stable mRNA [48] can be added after in vitro transcription using recombinant poly(A) polymerase [31–33].

The question is whether mRNA can be synthesized without a cap and poly(A) tail and still function when introduced into cells. For in vitro mRNA transcripts to function without a 5′ cap would require an alternative way to initiate protein synthesis. One way to do this is to include an internal ribosome entry site (IRES) in the 5′ region to initiate translation. Cells use IRESs to increase translation of certain proteins during mitosis and programmed cell death [49, 50]. IRESs are often used by viruses to ensure that viral translation is active when host translation is inhibited [51]. Although plus-strand RNA virus genomes that utilize IRESs to promote cap-independent translation are influenced by poly(A) binding proteins (PABP) and poly(A) status, transcription from other IRESs show much less dependence on the polyadenylation status [52]. The encephalomyocarditis viral (EMCV) IRES used in the vectors described here does rely on the conventional set of eukaryotic initiation factors (except eIF4E and intact eIF4G) [38], but it does not require PABP or 5′–3′ communication with the poly(A) tail at least during the first-round of initiation. Thus, using an IRES rather than a 5′ cap to initiate protein synthesis allows for removal of the poly(A) tail without significantly impairing protein synthesis. It should be possible, therefore, to produce uncapped and non-adenylated mRNA with an open reading frame downstream of an IRES that efficiently translates protein(s). This approach has not been used in either molecular biology applications or in vaccine or therapeutic drug production, however, because the un-capped 5′ and non-adenylated 3′ end are extremely sensitive to exonuclease-mediated degradation reducing mRNA stability.

One way to protect exogenously generated uncapped and non-adenylated mRNA from exonuclease degradation is to construct circular RNA. IRES-driven RNA vectors can be engineered to form circular RNAs lacking both a cap and poly(A) tail. Such circular RNAs do not have free ends that are vulnerable to exonucleases and thus showed an increased stability that resulted in extended duration of protein expression [53]. However, circular RNAs lack flexibility due to their rigid secondary structure and transfection of cells with exogenous circular RNA results in the activation of antiviral gene products such as OAS, PKR, and RIG-I which can initiate the cellular response against circular RNA [54].

An alternative method for protecting the terminal ends of mRNA lacking a cap and poly(A) tail is to include nucleotide hairpins at the 5′ and 3′ ends. A nucleotide hairpin is a pairing of complementary base pairs that is an essential secondary structure of RNA. It can guide RNA folding, determine interactions with ribozymes, protect mRNA from degradation, serve as a recognition motif for RNA binding proteins or act as a substrate for enzymatic reactions [55]. It has been shown that a 5′-terminal stem-loop structure can stabilize mRNA in different bacteria [39, 56] probably by preventing RNase E from interacting with the 5′ end of the message [39, 57–59]. Of note, it appears that the *location* of this stem-loop at, or very near, the 5′ and 3′ terminus is crucial to its stabilizing effect, whereas the *sequence* of this hairpin and its position relative to the ribosome binding site appears to have little effect. Up to two unpaired nucleotides upstream of the 5′ hairpin are tolerated without any reduction in mRNA stability, but the addition of 10–15 unpaired nucleotides of random sequence is as destabilizing as deletion of the 5′ hairpin [39]. A strong Shine–Dalgarno sequence near the 5′ end of the message in *E.coli* can recruit ribosomes and stabilize the message by blocking access of nucleases to degradative signals present in the naked mRNA [39, 60].

A role for terminal hairpin structures in eukaryotes has not been widely studied because such structures appear to be uncommon in metazoans. At the 5′ end, a terminal hairpin can interfere with cap-induced processes, and at the 3′ end, the majority of mRNAs are flanked by a polyadenylation signal followed by 10–30 downstream nucleotides and a poly(A) tail which makes the formation of a terminal hairpin unlikely [8, 11]. Non-polyadenylated mRNAs are rare in eukaryotes [61].

We hypothesized that we could protect both ends of the exogenous mRNA vector by adding stable hairpins during in vitro synthesis; this could stabilize the molecule and protect the mRNA from exonuclease degradation when delivered to target cells. However, this could be possible only with mRNA that does not depend on their ends to function. Including an IRES in the 5′ end to initiate translation would allow us to generate in vitro mRNA transcripts in a single step, bypassing the costly and time-consuming 5′ capping and 3′ poly (A) addition.

Using this strategy, we generated an effective mRNA transcript that had single hairpins at each terminal end that was the equal of conventionally constructed mRNA that contained a 5′ cap and a 3′ poly(A) tail. The use of triple instead of single hairpins at each end resulted in the highest reporter expression of any vector tested. Such mRNA structures are uncommon in eukaryotic cell, but they can be easily synthetized in vitro in a single step and then delivered as drugs or vaccines. In these experiments, we used the EMCV IRES, but other IRESs may potentially be used as well. However, each IRES may function differently depending on the vector, so results would need to be validated experimentally [62].

Exogenous mRNA used for therapy or vaccines often uses N1-methylpseudouridine (m1Ψ) instead of uridine to avoid the immune response and cytotoxicity induced by introducing mRNA into cells. This was based on the breakthrough studies from Kariko and co-workers, who showed that base modifications naturally found in human RNA such as pseudouridine, thiouridine, and 5-methylcytidine reduced the immunostimulatory potential of exogenously introduced RNA [63]. m1Ψ substantially out-performed all other modified bases studied leading to higher protein expression, more efficient translational repression in the presence of target microRNAs, and improved performance in cell culture [64–68]. Both approved COVID-19 mRNA vaccines use m1Ψ instead of uridine [46, 47]. In our studies, substituting m1Ψ for uridine in the vectors with triple hairpins on both sides improved eGFP expression by approximately 25%.

These results demonstrate that a 5′ cap and a 3′ poly(A) tail are not always required for the successful expression of exogenously generated mRNA in eukaryotic cells. The inclusion of a 5′ IRES is sufficient to initiate translation of the encoded protein. The inclusion of hairpins (single or triple) at each end protects the mRNA against degradation by exonucleases. This provides a potential method for rapidly generating exogenous mRNA using a single step, thus saving time and reducing cost.

## Data Availability

Additional data are available from the corresponding authors upon request.
